# Characteristics and Outcome of Obstetric Acute Kidney Injury in Pakistan: A Single-center Prospective Observational Study

**DOI:** 10.7759/cureus.3362

**Published:** 2018-09-26

**Authors:** Syed Rizwan A Bokhari, Faisal Inayat, Mah Jabeen, Zumar Sardar, Sara Saeed, Ayesha M Malik, Sabin Nasir, Amtul Zareen, Hafiz Ijaz Ahmad

**Affiliations:** 1 Nephrology, Tulane University School of Medicine, New Orleans, USA; 2 Internal Medicine, Allama Iqbal Medical College, Lahore, PAK; 3 Obstetrics and Gynecology, Pakistan Institute of Medical Sciences, Islamabad , PAK; 4 Neurology, Mayo Hospital King Edward Medical College, Lahore, PAK; 5 Obstetrics and Gynecology, Allama Iqbal Medical College, Lahore, PAK; 6 Nephrology, Allama Iqbal Medical College, Lahore, PAK

**Keywords:** obstetric acute kidney injury, sepsis, recovery, mortality, developing countries

## Abstract

Introduction

Acute kidney injury (AKI) continues to be a cause of increased morbidity and mortality in pregnant women. While studies have been conducted on the incidence and etiology of this complication, the outcomes of obstetric AKI have not been extensively investigated. The primary focus of this prospective observational study was to analyze the risk factors, etiologies as well as maternal and fetal outcomes of AKI in pregnant females in Pakistan.

Methods

A total of 56 patients with obstetric AKI were recruited. Patients were followed for a period of three months postpartum. The diagnosis and staging of AKI were based on the classification of the Acute Kidney Injury Network (AKIN).

Results

Fifteen patients were lost to follow-up and were excluded from the study. The mean age of the remaining 41 patients was 26±6 years. Twenty-two (54%) patients were multigravida, and 19 (46%) were primigravida. Twenty (48%) patients did not receive any antenatal care, 13 (31%) were visited by a traditional birth attendant, and only eight (19%) had adequate antenatal care by a gynecologist. Out of 41 patients, seven (17%) presented before 28 weeks, and 34 (83%) patients presented after 28 weeks of gestation. Four (10%) patients were found to be in stage I, four (10%) in stage II, and 33 (80%) patients in stage III AKI during hospitalization. The causes of AKI included sepsis in 32 (78%), intrauterine death in 24 (60%), postpartum hemorrhage in 17 (41%), shock in 15 (36%), pre-eclampsia/eclampsia in seven (17%), and coagulopathy in three (7%) patients. Twenty-eight (68.3%) patients received hemodialysis during the hospital stay. Three-month follow-up showed complete resolution of AKI in 14 (34.2%) patients, partial resolution in seven (17%), end-stage renal disease in 10 (24.4%), and death in 10 (24.4%) patients.

Conclusion

The present study indicates that a vast majority of patients with obstetric AKI require dialysis. Residual renal dysfunction and end-stage renal disease were common at the three-month follow-up. Incidentally, sepsis and intrauterine death were the leading causes in this study population. Increased awareness and appropriate obstetrical care may have a significantly positive impact on decreasing the morbidity and mortality in these patients.

## Introduction

Although the incidence of obstetric acute kidney injury (AKI) has significantly decreased in the developed world, it remains a serious public health problem in developing countries [1-3]. On the basis of the pathophysiology, development of obstetric AKI is traditionally considered bimodal as it is different for early and late parts of the gestational period as well as for the postpartum period [4]. During the first trimester of pregnancy, this condition is frequently reported secondary to septic shock. In the third trimester and immediate puerperium, it is found to be associated with pre-eclampsia/eclampsia, antepartum hemorrhage (APH), postpartum hemorrhage (PPH), puerperal sepsis, hemolytic uremic syndrome (HUS), disseminated intravascular coagulation, and hemolysis, elevated liver enzymes, and low platelet levels (HELLP) syndrome [5]. Prognosis of HUS and HELLP syndromes is poor in pregnant women and leads to a relatively higher mortality rate. Acute bilateral cortical necrosis is another deadly prognostic factor in obstetric AKI [6,7].

In regards to the outcome of this condition, complete renal recovery is commonly achieved if these patients receive an appropriate and timely management. Fewer patients may become entirely dependent upon dialysis due to inadequate initial resuscitation, longer intervals to reach an appropriate clinical setting, and consequently, significantly late initiation of dialysis [8]. Sibai et al. in their study highlighted renal outcomes in pregnant females depicting that bilateral renal cortical necrosis is found frequently in cases of abruptio placentae [9]; however, the occurrence of renal cortical necrosis and other irreversible renal changes in obstetric AKI remains unclear. Maternal mortality is another outcome of this syndrome. Maternal mortality rate increases with increasing severity of pregnancy-associated complications and it can range from approximately 13% to 24% in developing countries owing to obstetric AKI [8]. Fetal outcomes are also poor in pregnant women with AKI. It can result in late intrauterine death (IUD) of fetus and stillbirth. Furthermore, perinatal mortality is found to be notably high in neonates born to mothers with obstetric AKI [10]. Hence, obstetric AKI results in severe complications and higher maternal and fetal mortality rates.

The present study undertakes the analysis of numerous etiologies and outcome of obstetric AKI in patients admitted to a tertiary medical center in Pakistan. The preliminary form of the data was presented as an abstract (Abstract: Bokhari SRA, Ahmad HI, Zareen A, Saeed S, Nasir S, Malik A, Asif A. Outcomes of Obstetric Acute Kidney Injury at a University Medical Center of a Developing Country'', American Society of Nephrology Kidney Week, October 30 – November 04, 2012, San Diego, California).

## Materials and methods

This prospective, observational, single-center study was conducted at the Hemodialysis Center, Department of Nephrology and Hypertension, Jinnah Hospital, Allama Iqbal Medical College, Lahore, Pakistan. The research protocol was approved by the institutional review board. Informed written consent was obtained from all enrolled patients and their one close relative, which was in accordance with the principles of the Declaration of Helsinki as revised in Seoul in 2008. The study population included a total of 56 patients with obstetric AKI, recruited using non-probability convenience-type sampling. Exclusion criteria comprised of chronic kidney disease, history of hypertension, diabetes mellitus, history of renal calculi or the presence of small echogenic kidneys on ultrasonography. The patients were followed for three months postpartum.

Baseline demographic profile and clinical data, including age, educational status, parity, gestational age, mode of delivery, antenatal care, pre-eclampsia, eclampsia, abruptio placentae, placenta previa, antepartum or postpartum hemorrhage, symptoms of infection and urine output were recorded using a self-administered questionnaire. Moreover, data about blood transfusions, the time duration from the onset of AKI to the referral and initiation of dialysis, number of dialyses, urinalysis, biochemical and hematological tests, length of hospitalization, and changes in the maternal renal functions at the time of discharge were also recorded in a predesigned data record form.

Diagnosis of AKI was established according to the criteria of Acute Kidney Injury Network (AKIN) [11]. AKIN stage III patients were enrolled for hemodialysis, i.e., serum creatinine levels >4 mg/dL with an increase of more than 0.5 mg/dL or serum creatinine three times of baseline value or oliguria with urine output less than 0.3 mL/kg/hour for 24 hours or anuria for 12 hours. Renal recovery was analyzed in terms of renal function testing at the time of discharge from the hospital. Complete recovery was defined as the return of serum creatinine level to normal or <1.4 mg/dL. Return of serum creatinine value to ≥1.4 mg/dL after three months of AKI diagnosis was defined as partial recovery. The third category was a dialysis-dependent end-stage renal disease (ESRD) or all-cause mortality. Sepsis was defined according to the criteria of Systemic Inflammatory Response Syndrome (SIRS), set by the American College of Physicians [12]. Pre-eclampsia was considered as hypertension with proteinuria after 20 weeks of gestation and its progression to eclampsia in patients presenting with seizures.

Pregnancy outcome was categorized into abortion, preterm alive/dead fetus, and full-term alive/dead fetus. Delivery before 37 completed weeks of gestation was considered as preterm delivery. Data were analyzed using Microsoft Excel and Statistical Package for the Social Sciences (SPSS) 20.0 (IBM SPSS, Chicago, Illinois).

## Results

Fifteen patients were lost to follow-up and were excluded from the study. In 41 patients who completed the study, the mean age was 26±6 years. Twenty-two (54%) patients were multigravida, and nineteen (46%) were primigravida. Twenty (48%) patients did not receive any antenatal care, thirteen (31%) were visited by a traditional birth attendant, and only eight (19%) had adequate antenatal care by a gynecologist. Out of 41 patients with obstetric AKI, seven (17%) patients presented before while 34 (83%) presented after 28 weeks of gestation (Table 1).

**Table 1:** Baseline demographic characteristics of the study group (n=41). AKI, acute kidney injury; HSC, higher secondary school.

Characteristics		Mean ± SD/Percentage (n)
Age (years)		26±6
Antenatal care	Regular	19% (8)
	Irregular	31% (13)
	No	48% (20)
Number of pregnancies	Primigravida	46% (19)
	Multigravida	54% (22)
AKI at weeks of gestation	Before 28 weeks	17% (7)
	After 28 weeks	83% (34)
Educational status	Below primary	56%
	Below HSC	37%
	Above HSC	7%

**Figure 1:**
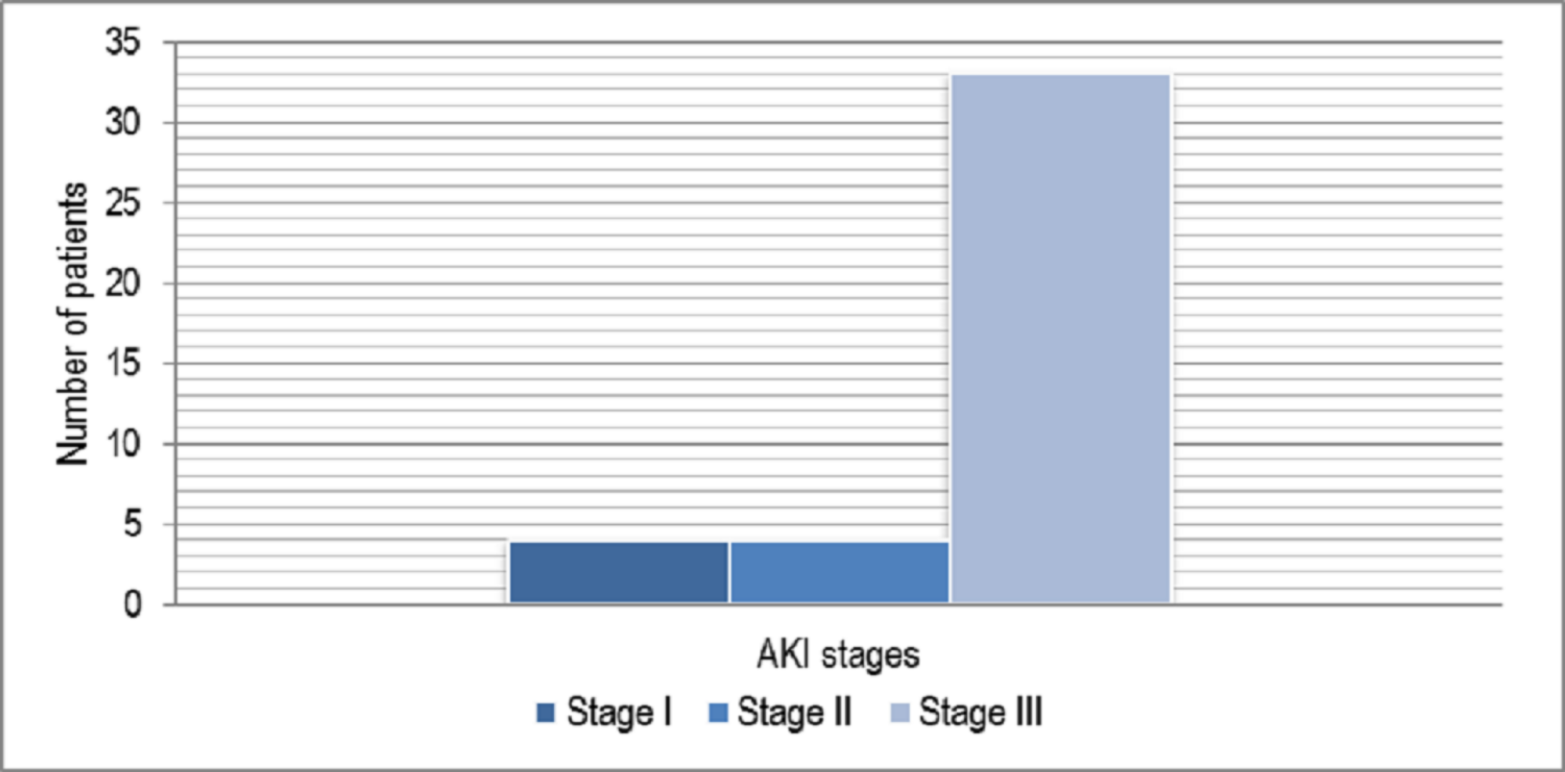
AKI in patients staged according to AKIN guidelines. AKI, acute kidney injury; AKIN, Acute Kidney Injury Network.

Four (10%) patients were found to be in stage I (serum creatinine level 0.5-2 times of baseline and urine output less than 0.5 mL/kg/hour for >6 hours), four (10%) patients in stage II (serum creatinine level 2 to 3 times of baseline and urine output <0.5 mL/kg/hour for >12hours), and 33 (80%) patients in stage III (serum creatinine >3 times of baseline and urine output <0.3 mL/kg/hour for 24 hours or anuria for 12 hours) of AKI during hospital stay. Patients requiring hemodialysis independent of their stage of AKI were also included in stage III (Figure 1).

The etiologies of AKI included sepsis in 32 (78%) patients, IUDs in 24 (60%), postpartum hemorrhage in 17 (41%), hemodynamic shock in 15 (36%), pre-eclampsia/eclampsia in seven (17%), and coagulopathy in three (7%) patients, and more than one of these factors were positive in multiple patients. Sepsis was the most common cause of AKI in our study group, and 78% of women had AKI due to this etiology. It was mainly associated with the unprofessional handling of traditional birth attendants and IUD. There was no significant increase in obstetric AKI among women with hypertensive disorders of pregnancy alone, which was attributable to the late presentation of the patients (Table 2).

**Table 2:** Etiologies of obstetric acute kidney injury among patients recruited in this study. Note: Multiple etiologies causing obstetric acute kidney injury were simultaneously present in our study population.

Etiology	Percentage (n)
Sepsis	78% (32)
Intrauterine death	60% (24)
Postpartum hemorrhage	41% (17)
Hemodynamic shock	36% (15)
Pre-eclampsia/eclampsia	17% (7)
Coagulopathy	7% (3)

Of the 41 patients, 28 (68.3%) patients received hemodialysis as the renal replacement therapy during their hospital stay. In the remaining patients with overt uremic features, hemodialysis was not initiated in three (7.3%) patients as one patient developed anoxic brain injury and consent for hemodialysis was not given by their surrogate health care decision makers in the remaining two patients with acute respiratory distress syndrome (ARDS). Ten (24.4%) patients were treated conservatively. Out of 28 patients, 25 (89%) patients required multiple sessions (range: 1-20) while three (11%) patients required a single session of hemodialysis. Fourteen (50%) patients became dialysis-independent and 10 (36%) patients remained dialysis-dependent at the end of the study, and these patients were categorized into the ESRD group.

**Figure 2:**
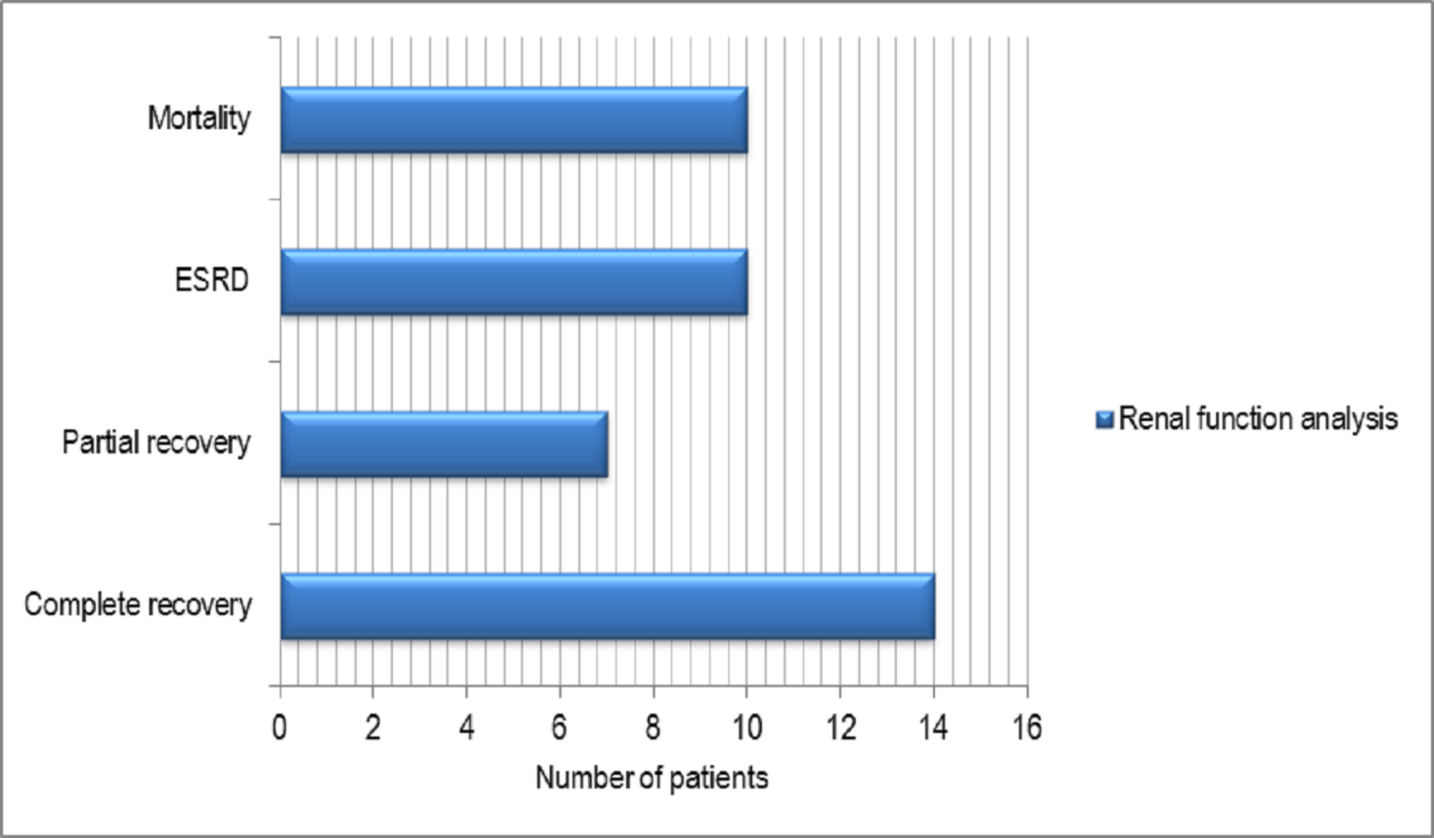
Renal function analysis at the end of the three-month follow-up period in this study.

At the three-month follow-up, complete resolution of AKI was observed in 14 (34.2%) patients, partial resolution in seven (17%), and ESRD in 10 (24.4%) whereas mortality occurred in 10 (24.4%) patients. Patients with partial recovery had renal dysfunction but were not dependent upon renal replacement therapy (Figure 2).

In regards to the pregnancy outcomes, results were generally poor. Maternal mortality rate was 24.4%, and the rate of fetal IUD was 60% in the present study.

## Discussion

The incidence of obstetric AKI is significantly higher in the developing regions of the world than in the developed countries. In a study conducted in Italy, Stratta et al. compared the incidence of obstetric AKI in four successive periods (1956-67, 1968-77, 1978-87, and 1988-94) and concluded that total incidence dropped down from 43% to 0.5% of total reported cases of AKI and from 1/3000 to 1/18,000 of total pregnancies in these years [13]. However, the incidence in developing countries is significantly high as compared to the incidence in developed countries [14]. The higher incidence was associated with substandard health facilities in peripheral areas resulting in poor antenatal care. The age of the mother is an essential parameter for a multitude of disorders. Similarly, several studies implicated a strong association between age and the onset of AKI in pregnancy. According to one report, the occurrence of obstetric AKI in women in the age group of 20-25 years is more frequent [15]. The mean age of the recruited patients in the current study was 26±6 years.

The etiologies of obstetric AKI are different in developed countries than in developing countries. Pre-eclampsia/eclampsia and PPH are the leading causes in developed nations and China [16]. However, 78% of women contracted AKI due to sepsis in the present study, making it the most common etiology. Previously, a study by Arora et al. from India also suggested sepsis as the leading cause of obstetric AKI with a maternal mortality rate ranging from 18% to 30% [17]. In these patients, sepsis is found to be associated with improper handling by traditional birth attendants, poor hygienic measures, non-sterilized interventions at the time of parturition, and IUD of the fetus. In this study, IUD was the outcome of pregnancy in 60% women. PPH was the cause of obstetric AKI in 41% women in our study group. This result is relatable to the findings of a study conducted by Ali et al. that depicted obstetric hemorrhage causing AKI in 58% of patients in their study population [18]. It was notable that pre-eclampsia/eclampsia caused obstetric AKI in 17% women in this study, which is relatively less common as compared to that in the developed world. This difference is primarily due to early detection and diagnosis of hypertensive disorders of pregnancy in the Western countries. In our region, patients present with later stages of pre-eclampsia/eclampsia where complications like obstetrical hemorrhage, shock, IUD, and stillbirth have already occurred.

The majority of the patients with obstetric AKI in our study group underwent hemodialysis as renal replacement therapy. Twenty-eight (68.3%) patients received hemodialysis during hospitalization; however, it could not be initiated in three (7.3%) patients. These results are comparable to the studies conducted previously by Ali et al. [18] and Ansari et al. [19] showing hemodialysis as renal replacement therapy in 76% and 71% patients of their study population, respectively. In this study, the outcome of hemodialysis was generally favorable showing that 50% of patients became dialysis-independent at the end of the three-month follow-up period.

Maternal mortality is a grave outcome of obstetric AKI. In our study, the overall mortality rate was 24.4% at the end of the three-month follow-up period, while among the participants undergoing hemodialysis, it was 14%. Our results are very close to the results of a few previous studies showing maternal mortality of 18% [18] and 26% [19]. However, a study conducted in South Africa demonstrated the maternal mortality rate as low as 5% [20]. A relatively higher rate of maternal mortality in developing countries like Pakistan is typically associated with the delay on the part of the patients and their attendants. Generally, in far-flung areas of Pakistan, obstetric and gynecological care is not up to the requirement, and it is a common trend among women to consult untrained traditional birth attendants. In the present study population, 19% of patients had regular visits to physicians and/or obstetricians, 31% were visited by traditional birth attendants, and unfortunately, 41% had received no antenatal care.

Fetal mortality rate was also remarkably high in our study. The rate of IUD was 60% owing to maternal etiologies. In developed countries, the fetal mortality secondary to this syndrome is about 44% [20], which is comparatively lower than our results. This difference is attributed to a relatively better antenatal and perinatal care in those countries. Dialysis dependency in mothers also results in higher incidence of fetal loss that further deteriorates the morbidity and, in turn, increases the risk of maternal mortality [21].

This study has several limitations, including i) shorter length of hospital stay, ii) fifteen patients could not complete the study, iii) it is notable that extended follow-up of the patients would have been of greater help in assessing the long-term impact of this condition on morbidity, mortality, and dialysis dependency, iv) renal biopsy was not performed in patients with partial recovery, v) specialized investigations like radioisotope renography should have been undertaken in patients showing delayed improvement for more than three weeks.

## Conclusions

This study highlights sepsis as the leading cause of obstetric AKI followed by IUD, PPH, pre-eclampsia/eclampsia, shock, and coagulopathy. Furthermore, substantial maternal mortality occurred due to sepsis and IUD. The current study also demonstrates that a vast majority of patients with obstetric AKI require hemodialysis as renal replacement therapy. Residual renal dysfunction and ESRD were frequently encountered at the three-month follow-up in our study group. Therefore, increased awareness and proper antenatal/obstetric care, especially in rural areas are warranted. These measures may help to reduce the incidence of obstetric AKI and associated maternal and fetal mortality rates in developing countries like Pakistan.
